# Low levels of antimicrobial resistance amongst canine staphylococci isolates from a remote First Nations community

**DOI:** 10.1016/j.onehlt.2026.101372

**Published:** 2026-02-21

**Authors:** Anna E. Sri, Kirsten E. Bailey, Amy W. Hii, Joan Malku Dhamarrandji, James Bayung Garrawitja, Joanne L. Allen, Rhys N. Bushell, Marc S. Marenda, James R. Gilkerson, Glenn F. Browning, Laura Y. Hardefeldt

**Affiliations:** a Asia-Pacific Centre for Animal Health, Melbourne Veterinary School, Faculty of Science, University of Melbourne, Parkville, VIC 3010, Australia; b National Centre for Antimicrobial Stewardship, Department of Infectious Diseases Melbourne Medical School and Melbourne Veterinary School, University of Melbourne, VIC 3010, Australia; c Yalu Aboriginal Corporation, Galiwin'ku, Northern Territory, Australia

**Keywords:** One health, Dogs, Aboriginal communities, First nations, Microbiology, Bacteriology, Epidemiology, Staphylococci, MRSA, MRSP, Coagulase negative staphylococci, Coagulase positive staphylococci, Stewardship, Antimicrobial resistance, Critically important antimicrobials, Treatment guidelines, High importance antimicrobials, Antibiotic, Companion animals, Rating system

## Abstract

Little is known about the ecology and resistance profiles of staphylococci on dogs in remote First Nations communities, nor how this might differ compared to isolates from other environments, people or animals. To investigate the prevalence, species diversity and proportion of antimicrobial resistance of staphylococci isolates in a dog population from a remote First Nations community in the Northern Territory, Australia, eighty-three dogs were sampled over two sampling periods in 2021 and 2022, using a house-to-house sampling methodology.

Nine species of staphylococci were identified, in addition to the recently reclassified Gram-positive species *Mammaliiococcus sciuri*. Methicillin-resistant *Staphylococcus pseudintermedius* and methicillin-resistant *Staphylococcus aureus* were not detected. Overall, resistance to antimicrobials, with the exception of penicillin, was low.

Results suggest current antimicrobial prescribing guidelines are appropriate for treating suspected staphylococcal infections in dogs in this community. To maintain the current low levels of resistance in staphylococci in this canine population, antimicrobial stewardship measures should extend beyond traditional stewardship interventions to acknowledge and address upstream influencing factors such as access to diagnostic tests, over-crowding, funding and workforce challenges.

## 1 Introduction

Resistance to antimicrobials in staphylococci is an emerging area of concern in companion animals, particularly in the coagulase positive staphylococci (CoPS), *Staphylococcus pseudintermedius* and *Staphylococcus aureus* [1], [2], [3]. Coagulase-negative staphylococci (CoNS) have also been recognised as potential pathogens, rather than commensals [4], [5] of humans and animals, and their role as a reservoir of transferable genetic resistance elements is also concerning [6], [7], [8], [9].

Staphylococcal isolates from Australian companion animals have been found to have lower frequency of resistance than from companion animals in other countries [8], [10], [11]. However, active and passive surveillance for resistance in companion animals is limited in Australia [12], [13]. The lack of long-term surveillance of antimicrobial use (AMU) and resistance impedes our ability to target antimicrobial stewardship (AMS) interventions and further extends the gap in knowledge around the ecology of antimicrobial resistance (AMR) in Australian animals. These difficulties are further compounded by the low rates of antimicrobial culture and susceptibility testing rates in animal health [10], [14], [15].

Active surveillance sampling methods that only identify isolates resistant to specific antimicrobials of importance to human health, such as methicillin, by use of selective media, do not provide a true representation of antimicrobial susceptibility. Studies using only this approach may also fail to detect species diversity within the bacterial population as this method only selects for resistant organisms.

Australia has a national antimicrobial importance rating system [16] and there are Australian-specific veterinary antimicrobial prescribing guidelines for numerous conditions affecting companion animals, including wounds, abscesses, and bacterial dermatitis [17]. However, these guidelines were developed based on information available from cities and regional areas, rather than from remote areas. Veterinarians practicing in remote Indigenous communities face additional challenges when attempting to diagnose and treat bacterial infections in animals. These include lack of access to in-house diagnostics, such as cytology and haematology, as well as extremely limited access to laboratory services to perform bacterial culture and susceptibility testing [18]. Many remote communities may also only have access to veterinary services intermittently for short periods of time, which does not allow regular follow-up of cases nor ongoing treatment with close supervision from a veterinarian, as would be considered the usual standard of care in most regional or urban areas of Australia [18], [19].

This study aimed to progress knowledge about the antimicrobial resistance profiles of staphylococci isolated from dogs in a remote Indigenous community and provide a basis for future initiatives to prevent AMR and evaluate AMS initiatives. Baseline data can be used to evaluate the feasibility of sampling from dogs in the community to act as sentinels of emerging AMR amongst the canine population and to evaluate the risk to public health from AMR in dogs. Active sampling of generally healthy animals performed in this study prevents reliance on culture and susceptibility data derived from passive surveillance in this and other similar populations. While increased passive surveillance data would be beneficial for understanding AMR in the remote context, at present it is too infrequently performed to provide significant insight into resistance patterns.

Only two studies have been reported relating to AMR from canine bacterial isolates in remote communities across Australia, one in regional New South Wales and one in the Kimberley region of Western Australia [20], [21], [22]. This is the first study of its kind conducted in the Northern Territory. It also uses a house-to-house sampling methodology and a quantitative method to assess antimicrobial susceptibility.

## 2 Methods

### 2.1 Sample population and context

Dogs were recruited from a community located on an island in a tropical region of the Northern Territory, Australia. All sampling was undertaken during the dry season. The dog population in the community at the time of sampling was estimated to be around 550–700 dogs, based on local council reports. There is annual variation in the population of dogs as new puppies are born and dogs die from infectious disease or because of accidents, in addition to the expected attrition attributable to age-related illnesses.

There was no permanent veterinary clinic in this community, and veterinary services were provided on 3–4 occasions per year for periods of a few days to a few weeks. Veterinary and animal care services in the community have been intermittent since 1977, and have varied in the level of service provision and training of the participants involved [23]. The majority of veterinary services in this community were focused on population control (desexing and euthanasia), as well as treatment and prevention of parasites. Anecdotally, administration of antimicrobials to dogs in the community may have increased over the last four years as a result of the detection and treatment of ehrlichiosis in dogs in the community, after this pathogen was first detected in Australia in mid-2020 [24].

Information collected during the study included dog breed, antimicrobial use in the last six months and desexing status.

### 2.2 Sample process

Sampling was undertaken on two separate occasions during the dry season, in June 2021 and August 2022. A house-to-house sampling methodology was used where the researchers visited each house in the community and made contact wherever people were at home. Householders were asked if they provided consent to sampling of their dog/s. Samples were collected from each animal using separate sterile plain rayon swabs (Cutiplast LP Italiana and Copan) moistened just prior to sampling with 0.9% saline. Samples from the oral mucosa, skin of the perineum and from any skin lesions or wounds were collected. Perineal samples were more commonly taken from small dogs and puppies that were used to being held by their owners. Swabs were not taken if the dogs resented sampling. Other reasons for not collecting samples included lack of owner consent (this occurred very rarely), owner not home at time of visiting, or dogs not at the premises at the time of visiting. None of the dogs sampled had received antimicrobials in the previous six months, according to their owners. The majority of dogs were kept as companion animals and seen as family members. Some were used for hunting and to provide security.

Samples were collected and kept in a cooler box, prior to being stored at 4 °C until the end of the sampling trip. At the end of the trip, samples were transported in a cooler box with ice packs from the community to the laboratory, which took up to twenty hours. As far as possible, no re-sampling of dogs was undertaken. This was confirmed by checking with the owners if their dog/s had been involved in the study, checking their name and description against previously sampled dogs, the lot number (address) sampled from and via scanning their microchip where possible.

### 2.3 Bacterial isolation

Each swab was inoculated onto sheep blood agar (SBA) (Thermofisher Scientific) and mannitol salt agar (MSA) (Edwards Group), and incubated overnight at 37 °C. In addition, swabs were placed in 3 ml of a 6.5% sodium chloride enrichment broth (Oxoid brain heart infusion broth in 2021 and heart infusion broth in 2022) and incubated at 37 °C overnight. The enrichment broth was then used to inoculate SBA and MSA plates, which were incubated overnight at 37 °C.

Up to 3 colonies were selected from each MSA plate based on morphological appearance on MSA and SBA plates, and sub-cultured onto SBA plates. All colonies from the SBA plates with a gross morphology consistent with that of staphylococci were Gram stained and Gram-positive cocci with catalase test positive were sub-cultured. All suspected staphylococcal isolates were then stored in LB broth containing 20% glycerol. The tube coagulase test and API (analytical profile index) ID 32 Staph kits (Biomerieux) were used to identify isolates to the species level. Antimicrobial susceptibility testing was performed following the VET01 Clinical & Laboratory Standards Institute (CLSI) broth microdilution method using Sensititre companion animal plates (Thermo Fischer Scientific). Plates were read at 18 and 24 h using the Sensititre ARIS 2× system for incubation and plate reading. The Sensititre plates included the following antimicrobials; amikacin, amoxicillin clavulanic acid, ampicillin, cefazolin, cefovecin, cefpodoxime, cephalothin, chloramphenicol, doxycycline, enrofloxacin, erythromycin, gentamicin, imipenem, marbofloxacin, minocycline, nitrofurantoin, oxacillin, penicillin, pradofloxacin, rifampicin, tetracycline, trimethoprim sulfamethoxazole, vancomycin.

### 2.4 Data analysis

CLSI breakpoints were used to classify isolates as susceptible or not. Confidence intervals for proportions were calculated using the Exact Method (Clopper-Pearson) using the binom.test function in R (version 4.1.2; R Core Team, 2021) and Fisher's exact test was performed for comparisons of proportions using the fisher.test function.

## 3 Results

Eighty-three dogs were sampled (27 in July 2021 and 56 in August 2022). Two of the dogs sampled were desexed the same day that they were sampled and received antimicrobials after sampling, consistent with the treating veterinarian's decisions. The dogs sampled were mostly mixed breed dogs with an indoor and outdoor lifestyle. Eight dogs sampled were from single dog households. The remaining dogs were from multi-dog households.

A total of 73 staphylococcal isolates were obtained from 43 of the 83 dogs. Isolates were from the oral cavity, perineum and wound samples (Table 1). Nine staphylococcal species were identified (Table 2), as well as the recently reclassified species *Mammaliicoccus sciuri*. One isolate was not identifiable to species level. Methicillin-resistance was not detected in any *S. pseudintermedius* isolates (prevalence 0%, 95% CI 0.0–0.044%]) or three *S. aureus* isolates (prevalence 0%, 95% CI 0.0–56.1%]). In five instances more than one staphylococcal species was isolated from the same swab, in four cases this was a CoNS and a CoPS. In one dog *S. hominis subspecies hominis* was isolated from the perineal swab, and *S. pseudintermedius* and *S. warneri* were isolated from the oral swab.

**Table 1:** Distribution of isolates by sampling site for dogs from which 1 or more *Staphyloccus* species were cultured (including *Mammaliiococcus sciuri).*

Site	No. swabs	No. dogs (prevalence %)	95% CI for prevalence
Oral mucosa	83	43 (55)	41.2–62.2%
Perineal skin	8	1 (13)	2.2–47.1%
Wound	2	1 (50)	9.5–90.5%
**Total**	**94**	**45**

**Table 2:** *Staphylococcus* spp. isolated from community dogs.

	Species	No. dogs	No. isolates from each site			Total
			Oral	Perineal	Wound	
Tube Coagulase positive	*S. pseudintermedius*	11	21	0	2	23
	*S. aureus*	3	3	0	0	3
Tube Coagulase negative	*S. simulans*	1	2	0	0	2
	*S. warneri*	1	1	0	0	1
	*S. hominis subsp hominis*	1	0	1	0	1
	*Mammaliicoccus sciuri*⁎	19	23	1	0	24
	*S. xylosus*	12	15	0	0	15
	*S. epidermidis 2*	1	1	0	0	1
	*S. hyicus*	1	1	0	0	1
	*S. saprophyticus*	2	2	0	0	2
**Total**		**43**⁎⁎				**73**

⁎ Formerly *Staphylococcus sciuri.*

⁎⁎ Some dogs had more than one isolate.

Of the twenty-three *S. pseudintermedius* isolates, thirteen (57%, 95% CI 34–77%) were susceptible to penicillin, and more than 90% were susceptible to all the other antimicrobials tested (Table 3). When compared to *S. pseudintermedius*, CoNS were more likely to be susceptible to penicillin (OR = 4.70, 95% CI [0.96, 31.76], *p* ≤0.05). Based on breakpoints for *S. pseudintermedius*, fewer CoNS were susceptible to third generation cephalosporins (Table 4).

**Table 3:**
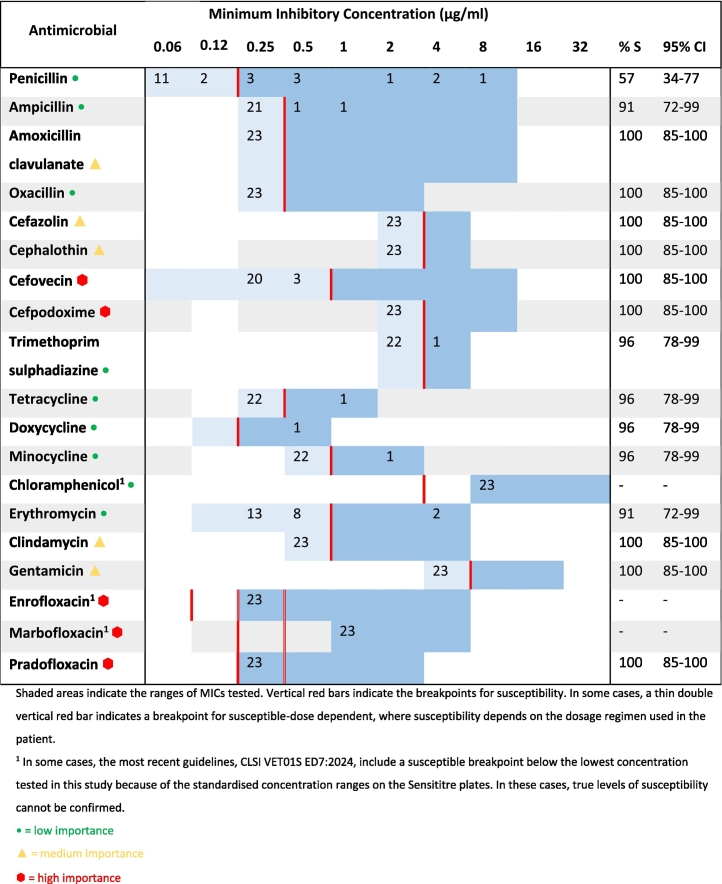
Minimum inhibitory concentration distributions for all *Staphylococcus pseudintermedius* isolates (*n* = 23) and proportions susceptible to each antimicrobial tested.

**Table 4:**
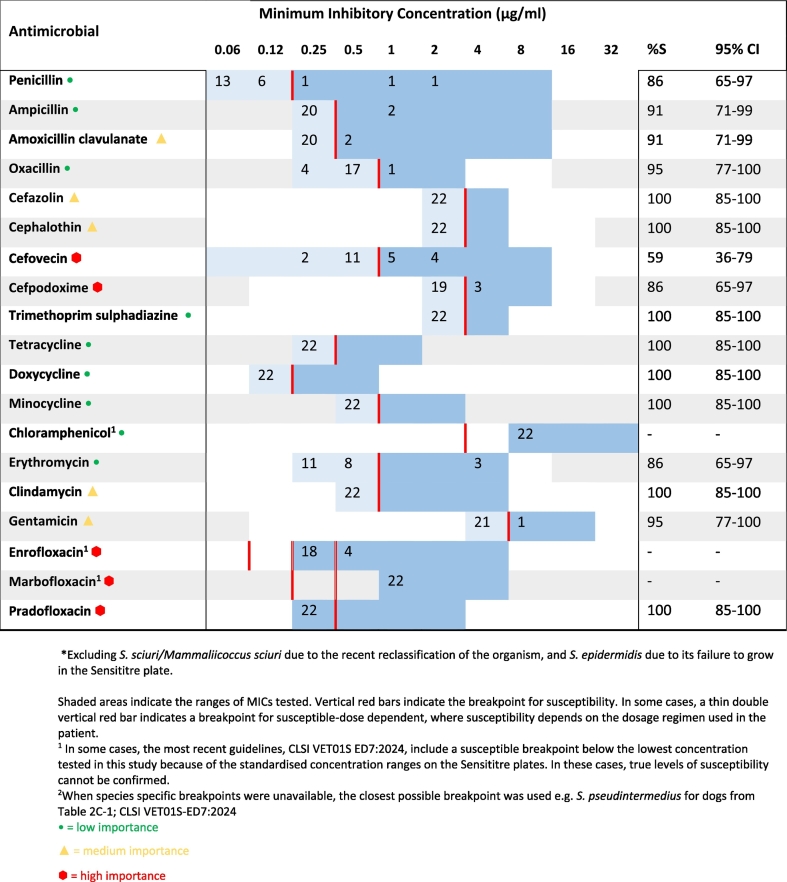
Minimum inhibitory concentration distributions for all coagulase negative staphylococcal isolates* (*n* = 22).

The MICs for the *M. sciuri* isolates were generally higher than those seen for *S. pseudintermedius*, *S. aureus* or other CoNS *Staphylococcus* spp. (Table 5). A single multi-drug resistant *S. pseudintermedius*, resistant to penicillin, trimethoprim/sulphadiazine, tetracyclines and erythromycin, but susceptible to ampicillin, was isolated from one of the wound samples. Resistance to high importance antimicrobials was not detected in any isolates.

**Table 5:**
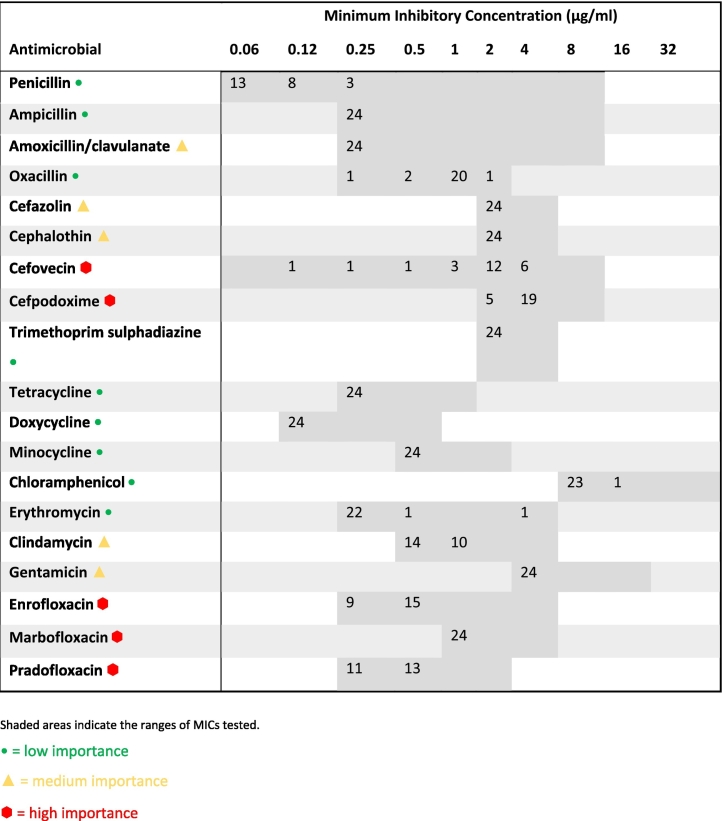
Minimum inhibitory concentration distributions for all *Mammaliicoccus sciuri* isolates (*n* = 24).

Results for amikacin, imipenem, nitrofurantoin, rifampicin and vancomycin were not reported in the antibiogram results tables (Table 3, Table 4, Table 5) to avoid misuse of active sampling data from healthy animals for veterinary treatment of sick animals. According to Importance Ratings and Summary of Antibacterial Uses in Human and Animal Health in Australia [16] these antimicrobials should not be used in dogs in Australia except in exceptional circumstances based on culture and susceptibility testing when there are no effective alternative agents. Similarly, none of these antimicrobials are currently recommended for use in dogs based on existing Australian-specific antimicrobial treatment guidelines.

## 4 Discussion

This study has provided information about the baseline rates of resistance in staphylococci in this population of dogs. Antimicrobial resistance in canine staphylococci does not appear to be an emerging One Health issue in this community. It suggests that, despite close contact between people and their dogs in this community, resistant staphylococci have not been transferred readily between healthy animals and people. Given reportedly high rates of MRSA in remote community human populations [25], if bacteria were being transferred between people and dogs, one may expect a higher proportion of methicillin-resistant isolates in the canine population. Other studies in Australia have detected higher rates of resistance amongst isolates from canine clinical cases [10], [26], [27]. The absence of methicillin-resistant *S. pseudintermedius* (MRSP) suggests there has not been high levels of antimicrobial use or movement of dogs from urban Australian into this dog population.

While AMR is a One Health issue, at present, AMR rates in staphylococci in this population of dogs are not high nor does there appear to be a significant risk of passing resistant staphylococci to humans from these dogs that are healthy yet carrying staphylococci in their mouths or on their skin, likely as a component of their normal flora. To more comprehensively study the zoonotic risk of AMR and also the risk to dogs from humans, it would be ideal to take samples from people, particularly dog owners, however this was not possible in this study. Similarly, further confirmation of identification through MALDI-TOF and molecular methods such as PCR and genomic sequencing were also not performed due to the higher cost and likelihood that these would not be sustainable surveillance tools in the long-term. Given the current low levels of resistance, in a resource-limited setting such as remote veterinary practice, these tests are unlikely to provide direct benefit to the canine population or the communities in which they live. In future, as costs reduce or if the risk of AMR increases, molecular methods including whole genome sequencing may be incorporated into AMR surveillance programs in this setting.

The mouth was chosen as a primary sampling site because previous studies have found that it was the most reliable site for isolation of staphylococci from dogs [28]. It was simple to take oral swabs from the dogs without restraint using positive reinforcement in the form of treats. This was preferable, as most of the dogs were not used to being restrained. The rate of isolation appeared low compared to some studies [20], [28], [29], but similar to others [30], with staphylococci isolated from 52% (*n* = 43) of dogs (95% CI 0.41, 0.62), and *S. pseudintermedius* from 13% of dogs (95% CI 0.08, 0.22). It is possible that this underrepresents the true prevalence because the difficult logistics of transporting samples to the laboratory may have reduced viability of the staphylococci on the swabs. However, it is evident samples can be collected in this remote setting and still yield results. Veterinarians working in remote settings should not be concerned about taking samples for culture and susceptibility testing provided that samples are packaged appropriately to ensure maintenance of low temperatures [31], [32] and there is good communication with the receiving laboratory. In-house diagnostic tests, such as cytology, may also help improve the appropriateness of antimicrobial prescribing, and can be performed even in remote settings. Cytology may currently be underutilized by veterinarians in remote settings because of time pressures and limited financial resources [33].

In general, susceptibility to low importance-rated antimicrobials was high, particularly for CoPS. The prevalence of susceptibility to penicillin amongst the *S. pseudintermedius* isolates obtained in this study was higher than amongst staphylococci isolated from a population of healthy dogs in Victoria (57% compared to 28%) [34]. The only multi-drug resistant *S. pseudintermedius* was isolated from a skin wound in an entire female dog with severe pyoderma. Contributing factors in this animal could have included previous treatment with antimicrobials (although this was not recalled by the owners), persistent infection due to immunosuppression from underlying disease, such as scabies or ehrlichiosis, or pregnancy, introduction by another animal, veterinarian or community member. A chronic infection may also provide the conditions for transfer of resistance elements from other bacterial species, including CoNS. Whether transfer of genetic elements from CoNS occurs, particularly in polymicrobial infections, requires further investigation. Evaluating culture and susceptibility data from a greater number of wounds or severe pyoderma cases could increase understanding about resistance patterns in bacteria isolated from clinical cases compared to these isolates of commensal staphylococci, which had low rates of resistance.

The absence of MRSP in this population was consistent with the results of a comparable study conducted in NSW. The authors of this study hypothesized that this was attributable to low rates of antimicrobial use, as was the case in the dog population in our study [20]. MRSA was not detected in this study and only three dogs carried *S. aureus*. In contrast, other similar studies have reported MRSA carriage rates of 2.6% [20], [22] and MSSA carriage rates of 4.4% [20]. Given that 83 dogs, approximately 10–15% of the estimated total community dog population, were sampled in our study, it is likely that, if MRSP or MRSA is present in this population, we can be 95% sure that the detectable prevalence is less than 4.4% for *S. pseudintermedius* isolates (95% CI 0.00–0.044) and less than 56% for *S. aureus* isolates (95% CI 0.00–0.56).

Further testing, including whole genome sequencing, to examine the *M. sciuri* isolates for the presence of the *mecA* methicillin-resistance gene, as well as other resistance genes, is needed to understand the role of this organism in the ecology of AMR in canine staphylococci. There are also no specific breakpoints available for *M. sciuri*, likely because this bacterial species is only rarely associated with clinical disease. This lack of breakpoints does make comparison of resistance between staphylococci and *M. sciuri* difficult.

While this study has helped in addressing gaps about AMR in remote dog populations and gaining an understanding of the risk of AMR to human health in this community, in the future, a focus on the health outcomes of the dogs, and appropriate treatment of sick or injured animals, is likely to be more cost effective in counteracting AMR. As resistance takes time to develop, robust monitoring of antimicrobial use (AMU) in this animal population in the future could provide a more feasible approach to AMS in this community rather than relying on further active sampling from healthy dogs, which is both time consuming and expensive.

### 4.1 Limitations

This is the first study of its kind conducted in the Northern Territory, and employing a house-to-house sampling methodology and a quantitative method to assess antimicrobial susceptibility. However, exact knowledge of the total population size from which samples are being collected would allow more accurate predictions of resistance levels across the animal population.

Practical challenges to sample collection such as the inability to take more swabs from the dogs due to temperament and time constraints may have impacted the results. Initially there was a target to take multiple oral swabs from each dog to increase the likelihood of bacterial isolation, however this was abandoned due to the increased time this added to the collection process and the impact this had on the dogs' compliance with the procedure. Similarly, it was not possible to take perineal swabs from most adult dogs without restraint yet restraining them was deemed to be an unnecessary risk to handlers and unnecessarily stressful to the dogs. The inability to reliably scan the dogs for a microchip prevented accurate identification of the dogs. More accurate identification would have confirmed that re-sampling did not occur or at least would have allowed better monitoring for re-sampling events. It also may have allowed some correlation of potential antimicrobial usage with veterinary records, although it was noted at the time of sampling that veterinary records for some dogs sampled were months to years out of date or there were no records available.

There was variable time between collection of the samples and bacterial isolation at the laboratory. Although isolation rates did not appear to vary significantly between enriched and non-enriched samples, consistent recording of the time of sample collection and subsequent isolation of bacteria may have enabled understanding of the length of time that a sample can remain viable for subsequent bacterial isolation at refrigeration temperatures.

The lack of wound samples prevented understanding of resistance patterns from clinical cases and may have underestimated the prevalence of staphylococci carriage and MRSP carriage. Similarly the low isolation rates of *S. aureus* meant that the prevalence could not be determined to a high degree of accuracy.

## 5 Conclusion

This study has increased understanding about the dynamic and diverse nature of carriage and colonisation with staphylococci in a canine population in a remote Indigenous community and highlighted how much is unknown about the ecology of staphylococci from a One Health perspective. A high level of susceptibility was detected to antimicrobials with a low-importance rating [16]. The successful use of this study's methodology may increase the confidence of veterinarians to undertake sampling for culture and susceptibility testing in future or undertake similar trials involving clinical cases. Reference to current antimicrobial prescribing guidelines for empirical treatment is also recommended, given the low levels of resistance detected in staphylococcal isolates in this dog population. This study was carried out with the understanding that AMR is a socio-political problem, not just a health issue. To maintain current low levels of resistance in staphylococci in the canine population more research and actions may need to focus on upstream social, economic, cultural, and legal conditions the underlie health inequalities and have been shown to drive and maintain AMR [35]. For example, improving access to veterinary care, ensuring safe and culturally appropriate housing and reducing socio-economic inequality.

## CRediT authorship contribution statement

**Anna E. Sri:** Conceptualization, Data curation, Formal analysis, Investigation, Methodology, Project administration, Visualization, Writing – original draft, Writing – review & editing. **Kirsten E. Bailey:** Conceptualization, Supervision, Writing – review & editing. **Amy W. Hii:** Methodology. **Joan Malku Dhamarrandji:** Conceptualization, Data curation. **James Bayung Garrawitja:** Conceptualization, Data curation. **Joanne L. Allen:** Data curation, Methodology. **Rhys N. Bushell:** Data curation, Methodology. **Marc S. Marenda:** Methodology. **James R. Gilkerson:** Conceptualization, Supervision, Writing – review & editing. **Glenn F. Browning:** Conceptualization, Writing – review & editing. **Laura Y. Hardefeldt:** Conceptualization, Funding acquisition, Supervision, Writing – review & editing.

## Informed consent statement

Informed consent was obtained from the owners of all dogs in the study.

## Declaration of competing interest

The researchers declare that they have no known competing financial interests or personal relationships that could have appeared to influence the work reported in this paper.

## Data Availability

Data will be made available on request.
